# Isolation and Differentiation of Mesenchymal Stem Cells From Broiler Chicken Compact Bones

**DOI:** 10.3389/fphys.2018.01892

**Published:** 2019-01-22

**Authors:** Roshan Adhikari, Chongxiao Chen, Elizabeth Waters, Franklin D. West, Woo Kyun Kim

**Affiliations:** ^1^Department of Poultry Science, University of Georgia, Athens, GA, United States; ^2^Regenerative Bioscience Center, University of Georgia, Athens, GA, United States; ^3^Department of Animal and Dairy Science, University of Georgia, Athens, GA, United States

**Keywords:** mesenchymal stem cells, isolation, broiler, biological characteristics, pluripotency

## Abstract

Chicken mesenchymal stem cells (MSCs) can be used as an avian culture model to better understand osteogenic, adipogenic, and myogenic pathways and to identify unique bioactive nutrients and molecules which can promote or inhibit these pathways. MSCs could also be used as a model to study various developmental, physiological, and therapeutic processes in avian and other species. MSCs are multipotent stem cells that are capable of differentiation into bone, muscle, fat, and closely related lineages and express unique and specific cell surface markers. MSCs have been isolated from numerous sources including human, mouse, rabbit, and chicken with potential clinical and agricultural applications. MSCs from chicken compact bones have not been isolated and characterized yet. In this study, MSCs were isolated from compact bones of the femur and tibia of day-old male broiler chicks to investigate the biological characteristics of the isolated cells. Isolated cells took 8–10 days to expand, demonstrated a monolayer growth pattern and were plastic adherent. Putative MSCs were spindle-shaped with elongated ends and showed rapid proliferation. MSCs demonstrated osteoblastic, adipocytic, and myogenic differentiation when induced with specific differentiation media. Cell surface markers for MSCs such as CD90, CD105, CD73, CD44 were detected positive and CD31, CD34, and CD45 cells were detected negative by PCR assay. The results suggest that MSCs isolated from broiler compact bones (cBMSCs) possess similar biological characteristics as MSCs isolated from other chicken tissue sources.

## Introduction

Global production of chicken meat has dramatically increased, and the consumer demand for high quality poultry meat has continuously risen in recent years (Scanes, 2007; Mir et al., 2017). However, genetic selection for rapid growth and high feed efficiency causes skeletal disorders, excess fat accumulation, and muscle degeneration in broilers, which are important health and economic issues for the poultry industry (Velleman et al., 2003; Fleming, 2008; Kim et al., 2011; Fornari et al., 2014). Thus, identifying innovative methods to minimize these issues in broilers is important for the sustainability of poultry production. Studies on chicken mesenchymal stem cells (MSCs) can provide critical insight for skeletal development, muscle growth, and fat accumulation in poultry. MSCs are multipotent, plastic adherent cells that have been isolated from several species, including human, mouse, rat, dog, cat, sheep, and chickens (Crigler et al., 2007; Dumas et al., 2008; Kar et al., 2016; Kumar et al., 2016; Li et al., 2016). The ability of MSCs to differentiate into lineage specific tissue types (Pittenger et al., 1999; Freeman et al., 2015), their homing potential (Sohni and Verfaillie, 2013), and tissue repair potential (Melo et al., 2016) has generated increasing interest in utilizing MSCs to study mechanisms in basic biology, as well as clinical therapeutics. Chicken MSCs have significant agricultural applications as they can be utilized to better understand the mechanisms that drive osteogenic, myogenic, and adipogenic differentiation and potentially be used to identify novel compounds that drive these processes to the more desirable muscle and bone phenotypes. MSCs derived from bone marrow were first described as fibroblast-like colony forming cells, which maintained differentiation potential, and proliferation (Friedenstein et al., 1987). The Mesenchymal and Tissue Stem Cell Committee of the International Society for Cellular Therapy (ISCT) has defined these minimal criteria for MSCs: (a) the capacity to adhere to a plastic surface under standard culture conditions, (b) have a multilineage differentiation property, and (c) display the presents and absence of surface antigens (Dominici et al., 2006).

One of the major challenges of isolating chicken MSCs utilizing bone marrow from non-compact bone sources is the requirement of multiple purification steps to remove non-MSC cell types (e.g., hematopoietic stem cells, blood cells) (Khatri et al., 2009). These purification approaches often lead to cytotoxicity, changes in functionality of MSCs, and low MSC yields (Wee et al., 2013). However, there are no reports of MSCs isolated from compact bones of the chickens, which may overcome these limitations as there are fewer contaminating cell types (Pittenger et al., 1999). There are two main stem cell populations that are the main residents of bone marrow: hematopoietic stem cells and MSCs (Pittenger et al., 1999; Zhu et al., 2010). One significant drawback of isolating MSCs from bone marrow is the contamination of blood cells and hematopoietic stem cells. Various techniques have been used to purify or enrich the MSC population isolated from bone marrow including preferential attachment to culture plastic, density gradient centrifugation (Yamamoto et al., 2015), use of ficole to filter out blood cells, antibody-based cell sorting (Van Vlasselaer et al., 1994; El-Sayed et al., 2015), low and high density culture techniques (Eslaminejad and Nadri, 2009), and frequent media change (Soleimani and Nadri, 2009). However, these methods used to purify MSCs isolated from bone marrow have a number of negative effects. Use of preferential attachment to cell culture plates could yield phenotypically and functionally heterogenous cell population (Tremain et al., 2001). Use of immune depletion techniques downregulated many genes involved in cell proliferation and cell cycle progression (Baddoo et al., 2003). Exposure of MSCs isolated from immunodepleted cells to insulin-like growth factor or leukemia inhibitory factor reversibly inhibited the ability of cells to differentiate into adipocyte, chondrocyte and osteoblasts *in vitro* (Baddoo et al., 2003). Use of low density culture yielded only about 27 fibrobalstoid colonies of 5 or more cells from a total of 200 culture disc (Wang and Wolf, 1990). Cell sorting approaches to isolate multi-lineage MSCs from hematopoietic cells resulted in reduced clonogenicity and limited osteogenic potentials in isolated MSCs (Van Vlasselaer et al., 1994). Isolation of MSCs from compact bones could be an easy and economic isolation technique which can avoid the use of other purification techniques during isolation and reduce the chances of hematopoietic cells contamination in the isolated cultures (Guo et al., 2006; Zhu et al., 2010).

In this study, we present for the first time, an effective, simple, and economical method for isolation and characterization of MSCs from compact bones (cBMSCs) of day old chickens. cBMSC are a robust and highly proliferative cell population that meet the ISCT MSC criteria. These cells open the door for future *in vitro* studies of critically important osteogenic, adipogenic, and myogenic pathways in avian species and for the identification of novel bioactive nutrients and molecules which promote skeletal health, muscular growth, and efficient feed utilization in poultry.

## Materials and Methods

### Ethics Statement

All experiments were performed in accordance with the guidelines for the use of animal in research as stated by the Institutional Animal Care and Use Committee at the University of Georgia. The protocol was approved by the Institutional Animal Care and Use Committee at the University of Georgia.

### Isolation of cBMSCs

**Figure 1:**
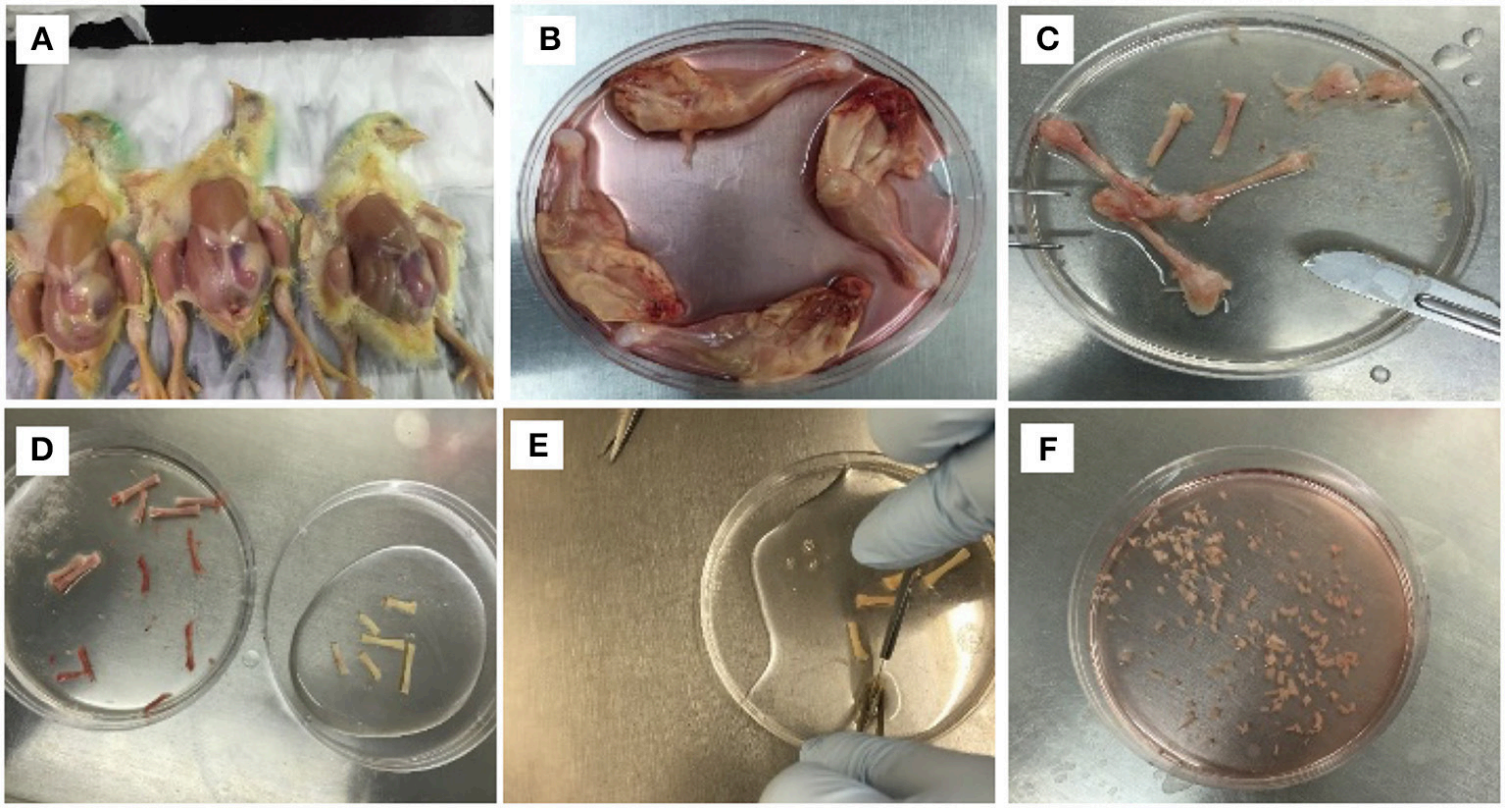
Isolation procedure of MSCs from compact bones of chick (cBMSCs). **(A)** Chicks were soaked in alcohol for leg dissections **(B)** and legs dissected and soaked in DMEM with 10% FBS. **(C)** Muscles were separated to obtain femur and tibia. **(D)** Epiphysis was dissected, and bone marrow was flushed with PBS containing 2% FBS. **(E)** After washing, the bones appeared white in color. **(F)** Bones were chopped in 1–3 mm^3^ and digested in digestion media.

cBMSCs were isolated by using a modified approach of the previously described methods in human trabecular and murine compact bones (Tuli et al., 2003; Zhu et al., 2010). Femurs and tibia bones from both legs were obtained from the day-old chicks after cervical dislocation. The birds were soaked in alcohol for 2 min after cervical dislocation. Legs were removed from hip joint and metacarpal (Figures 1A–C). Dissected legs were kept in Dulbecco's Modified Eagle's medium (DMEM) (Mediatech Inc.,VA, USA) containing 10% Fetal Bovine Serum (FBS) (Mediatech Inc.,VA, USA), 100 U/mL penicillin, 100 μg/mL streptomycin, and 0.292 mg/mL L-glutamine (Thermo Fisher Scientific, MA, USA) until connective tissues and muscles were completely removed. Muscles and connective tissues around tibia and femurs were removed immediately using a scalpel and micro-dissecting scissors in a bio-safety cabinet (Figure 1C). The cleaned tibia and femurs were placed in washing buffer containing Phosphate-Buffer Saline (PBS) (Mediatech Inc., VA, USA) and 2% FBS. The epiphysis of the bones were removed to expose the bone marrow cavity. Bone marrow inside the bone was flushed four times with washing buffer in a syringe to remove the bone marrow and hematopoietic cells adhered to the compact bones (Figure 1D). The bones were cracked with a scalpel and washed three more times with washing buffer to make sure that all the bone marrow cells were washed. The bones appeared whitish in color after the wash (Figure 1D). The bones were transferred to new cell culture dishes with 5 ml of digestion media (DMEM containing 100 IU/ml penicillin and 100 ug/ml streptomycin, 0.25% collagenase (Sigma-Aldrich, MO, USA), and 20% FBS). The bones were chopped to smaller fragments of about 3 mm^3^ (Figures 1E,F). Bone fragments were suspended in a 50-ml tube that contained digestion media. The bone fragments were digested in a shaking water bath for 60 min at 37°C at 180 rpm. The digestion media containing bone fragments were filtered with 40 μm sterile filter. Bone fragments in the filter were rinsed with 5 ml of 10% DMEM. Filtered contents were centrifuged at 1,200 rpm for 10 min. The supernatant was discarded and the cell pellet was disrupted with 20 ml 10% DMEM, and cells were plated in two 100-mm cell culture dishes. Cultures were incubated at 37°C in a humidified incubator containing 5% CO_2_. Half of the media was replaced by a fresh media at 12 h, complete media was changed at 24 h to remove the non-adherent cells. After that, media was changed once every 2–3 days. These cells were labeled as P0. Once the cells reached 95% confluently, the cells were washed twice with 5 ml 1X PBS, dissociated with 0.1% Trypsin-EDTA (Mediatech Inc., USA) for 2 min and subcultured at a ratio of 25,000 cells/ cm^2^ in 100-mm cell culture dishes. This passage was marked as P1, subsequent cultures were named as P2, P3, P4… Pn consecutively. P4 cells were used for cBMSCs differentiation experiments.

### cBMSCs Growth and Morphology Observation

cBMSCs were observed daily to understand their morphology and growth characteristics. Cells were observed under a microscope daily, and pictures of cells were taken at different time points. Cells were observed for morphological features and capacity to adhere to plastic plates.

### Colony Forming Unit-Fibroblast (CFU-F) Assay

Self-renewal capacity and colony forming potential in isolated MSCs were evaluated by plating cells at low densities in 100-mm cell culture dishes. Colonies formed from single cells were counted at P4 and P8. At both passage, 25 cells/cm^2^, 50 cells/cm^2^, and 75 cells/cm^2^ were plated in 100-mm cell culture dishes and incubated for 10 days. On day 10, cells were fixed and stained with 1% crystal violet (Sigma-Aldrich, MO, USA) in 100% methanol for 30 min. Colonies were defined as more than 50 fibroblastic cells in a cluster. The number of colonies were scored, and microscopic pictures were obtained.

### Growth Kinetics

Cells at P2, P4, and P8 were dissociated by 0.25% trypsin and plated in 24-well plates with a density of 1 × 10^4^ cells /ml. The number of cells were counted by a viability detection method using a trypan blue exclusion test on 4 wells daily for 7 days. Each well was counted three times, and mean value was calculated. Growth curves were plotted for each passage. Population doubling time (PDT) was calculated by formula reported by Aliborzi et al. (2016). PDT was calculated using the formula PDT = T ln2/ln (Xei/Xbi), where T means incubation time in hours, Xbi is the number of cells at the beginning of the incubation and Xei corresponds to the number of cells at the end of incubation.

### Multilineage Differentiation of cBMSCs *in vitro*

#### Osteogenic Differentiation

cBMSCs at P4 were seeded at a density of 20,000 cells /cm^2^ in 24-well plates for Alizarin Red (Gregory et al., 2004), Alkaline Phosphate (ALP) (Parhami et al., 1997), and Von Kossa stain (VK) (Parhami et al., 1997), and in 6-well plates for measurement of osteogenic gene regulation. Cells were cultured in basal media containing DMEM, 10% FBS, 100 IU/ml penicillin, and 100 μg/ml streptomycin until 90% confluent. On confluency, cells were treated with osteogenic media (OM) containing DMEM with 10^−7^M dexamethasone (DXA) (Sigma-Aldrich, MO, USA), 10 mM β-glycerophosphate (Sigma-Aldrich, MO, USA), 50 μg/ml ascorbate (Sigma-Aldrich, MO, USA), and 5% FBS for osteogenic induction. Cells cultured in DMEM basal media with 10% FBS were used as negative control. Fresh media was replaced with the old media in the culture plate in every 2–3 days. Cells were stained with Alizarin Red and VK for detection of mineralization and ALP for detection of osteogenic differentiation on 7 and 14 days of treatment. Cells plated in 6-well plates were harvested at 72 h for osteogenic gene expression analysis using Quantative Reverse Transcription Polymerase Chain Reaction (qRT-PCR). ALP, Runt-related transcription factor 2 (RUNX2), Bone Morphogenetic Protein (BMP2), Bone Sialoprotein (BSP), and Bone Gamma-Carboxyglutamate Protein (BGLAP) were analyzed to detect osteogenic differentiation of cBMSCs.

### Adipocyte Differentiation

cBMSCs at P4 were placed at a density of 20,000 cells/cm^2^ in 24-well plates and 6-well plates. At confluency, cells were treated with adipogenic cocktail (DMI containing 500 nM dexamethasone, 0.5 mM 3-isobutyl-1-methylxanthine and 20 mg/mL insulin) (Cedarlane, NC, USA) + 300 μM Oleic acid. The induced cells were observed under an inverted microscope for fat vacuole deposition. After 96 h of treatment with adipogenic media, cells cultured in 24-well plates were harvested and stained with Oil Red O using a procedure described by Kim et al. (2009). Cells treated in 6-well plates were harvested at 48 h for adipogenic gene expression using qRT-PCR. Key adipogenic gene, PPARγ, FABP2, c/EBPα, and c/EBPβ were measured for adipogenic differentiation of cBMSCs.

### Myogenic Differentiation

cBMSCs were cultured in 6-well plates at a density of 20,000 cells/cm^2^ and treated with myogenic medium (MM) containing DMEM, 5% horse serum (Thermo Fisher Scientific, MA, USA), 50 μM hydrocortisone (Sigma-Aldrich, MO, USA), and 0.1 μM dexamethasone when confluent. Cells cultured in 6-well plates were harvested after 72 h for key myogenic gene expressions such as MyoD, Pax7, Myf5, and Myogenin using qRT-PCR (Quantative Reverse Transcription Polymerase Chain Reaction to detect multilineage gene expression).

Total RNA was extracted from harvested cell with QIAzol Lysis reagent (Qiagen, MD, USA) following the manufacturer's protocol. Two micrograms of RNA was reverse-transcribed to make cDNA using High-Capacity cDNA Reverse Transcription Kits (Thermo Fisher Scientific, MA, USA). Template, multiscript reverse transcriptase enzyme, random primers, dNTPs, and buffers were subjected to thermocycling at 25°C for 10 min, 37°C for 120 min, and 85°C for 5 min in a veriti 96-well thermal cycler (Thermo Fisher Scientific, MA USA). Specific primers were used to quantify the osteogenic, myogenic, and adipogenic mRNA expression in harvested cells by qRT-PCR. iTaq^TM^ Universal SYBR Green Supermix (Bio-Rad, CA, USA), primers and cDNA templates were subjected to qRT-PCR at 95°C for 10 min, followed by 40 cycles of 95°C for 15 s, annealing temperature was variable according to primers for 20 s, and 72°C for 15 s, followed by 95°C for 15 s and a melt curve stage in a StepOne^TM^ Real-Time PCR machine (Thermo Fisher Scientific, MA, USA). All samples were prepared in duplicated wells in 96-well plates. Relative abundance of mRNA between the samples were analyzed by the ΔΔCT method. GAPDH was used as a housekeeping gene. The values were reported as fold changes of the mRNA expression of the target genes in differentiation groups compared to the control group. Primers for each gene were designed and checked for target identity using the National Center for Biotechnology Information (NCBI). Primers used for qRT-PCR assays and their sequences are presented in Table 1.

**Table 1:** List of primers used in the study.

**Gene name**	**Primer sequence (5^**′**^−−−3^**′**^)**	**Product length (bp)**	**Annealing temperature (^**°**^C)**
GAPDH^a^	Fwd: GCTAAGGCTGTGGGGAAAGT	116	55
	Rev: TCAGCAGCAGCCTTCACTAC	
FABP4^a^	Fwd: TGCTGGGCATCTCAATCACA	106	57
	Rev: GCATTAGTCAGAACGGGCCT		
PPARγ^a^	Fwd: TGAATGTCGTGTGTGTGGGG	229	55
	Rev: GCATTCGCCCAAACCTGATG		
C/EBPα^a^	Fwd: CCTACGGCTACAGAGAGGCT	205	55
	Rev: GAAATCGAAATCCCCGGCCA		
C/EBPβ^a^	Fwd: CCGCTCCATGACCGAACTTA	204	55
	Rev: GCCGCTGCCTTTATAGTCCT		
Col1A2^e^	Fwd: AGAAAGGAATCCAGCCCAAT	238	58
	Rev: ACACCTGCCAGATTGATTCC		
BMP2	Fwd: TGCTGTTGCTCTCAAAGGCT	300	57
	Rev: CTGTGCTTTCTGCCTGGAAGT		
BSP	Fwd: GGAACAGGGAGTCAGCAAGG	156	57
	Rev: TGCAGGGTGAAATGAAGCTCT		
BGLAP^a^	Fwd: GACGGCTCGGATGCTCGCAG	226	55
	Rev: CAGACGGGGCCGTAGAAGCG		
MyoD^f^	Fwd: CAGCAGCTACTACACGGAATCA	102	57
	Rev: GGAAATCCTCTCCACAATGCTT		
Myogenin^d^	Fwd: AGCAGCCTCAACCAGCAGGA	179	58
	Rev: TCTGCCTGGTCATCGCTCAG		
Pax7	Fwd: AGGCTGACTTCTCCATCTCTCCT	156	57
	Rev: TGTAACTGGTGGTGCTGTAGGTG		
Myf5	Fwd: GAGGAACGCCATCAGGTACATC	126	57
	Rev: ACATCGGAGCAGCTGGAGCT		
CD 29^c^	Fwd: GAA CGG ACA GAT ATG CAA CGG	300	60
	Rev: TAGAACCAGCAGTCACCAACG		
CD34^c^	Fwd: GTGCCACAACATCAAAGACG	239	60
	Rev: GGAGCACATCCGTAGCAGGA		
CD45^b^	Fwd: CACTGGGAATCGAGAGGAAA	574	55
	Rev: CTGGTCTGGATGGCACTTTT		
CD90^b^	Fwd: GGTCTACATGTGCGAGCTGA	471	56
	Rev: AAAGCTAAGGGGTGGGAGAA		
CD44^c^	Fwd: CATCGTTGCTGCCCTCCT	134	55
	Rev: ACCGCTACACTCCACTCTTCAT		

a *Regassa and Kim (2013)*.

b *Khatri et al. (2009)*.

c *Bai et al. (2013)*.

d *Gabriel et al. (2003)*.

e *Usui et al. (2008)*.

f *Sławinska et al. (2013)*.

### PCR for Cell Surface Markers

Cells were plated in 6-well plates and harvested when confluent at P2, 4, 6, and 8 to analyze cell surface markers. RNA was isolated from the harvested cells, and cDNA was transcribed following the method described above. Synthesized cDNA was subjected to PCR amplification using DreamTaq Green PCR Master Mix (Thermo Fisher Scientific, MA, USA) following manufacturer's protocol. Samples were subjected to thermocycling at 95°C for 3 min, followed by 40 cycles of 95°C for 30 s, the annealing temperature of primers for 30 s, and 72°C for 30 s, followed by 72°C for 7 min. PCR products obtained were separated by 1.5% agarose gel electrophoresis to visualize the bands detected using SYBR Safe DAN gel stain (Thermo fisher Scientific, MA, USA). mRNA expression of cell surface markers, CD44, CD29, CD45, CD90, CD105, and CD34, was used to characterize cBMSCs population at P2, P4, and P8. GAPDH was used as an internal control. Primer sequences for cells surface markers such as CD29, CD34, CD44, CD71 were used to characterize cBMSCs as reported (Khatri et al., 2009; Bai et al., 2013). Primers for each gene were designed and checked for target identity using the National Center for Biotechnology Information (NCBI). Primers used for PCR assays and their sequences are presented in Table 1.

### Immunocytochemistry

Cells were plated onto glass four-chamber slides (BD Bioscience, San Jose, CA, http://www.bdbiosciences.com) and fixed with 4% paraformaldehyde for 15 min. Antibodies were directed against CD44 (1:100; Bio Rad) and CD45 (1:100; SouthernBioTech). Primary antibodies were diluted in blocking solution and incubated for 1 h at room temperature. Primary antibodies were detected using secondary antibodies conjugated to Alexa Fluor 488 (1:1,000; Life Technologies) and incubated for 1 h at room temperature. Cells were then washed before mounting with Prolong Gold with DAPI (Life Technologies). Cell observations were made using the Olympus Ix81 (Olympus, Tokyo, http://www.olympus-global.com) with Disc-Spinning Unit and Slide Book Software (Intelligent Imaging Innovations, Santa Monica, CA, http://www.intelligent-imaging.com). Quantification was performed by imaging 5 random fields for each antibody in triplicate and utilizing the cell counter plugin on ImageJ 1.0 software (Schneider et al., 2012).

### Statistical Analysis

Statistical analysis was performed using the general linear model procedure of the Statistical Analysis System (SAS) software (SAS institute, 2011). Mean separation test was conducted using Tukey test and *P* ≤ 0.05 was considered as statistically significant among the groups.

## Results

### Putative cBMSCs Show Morpholgy and Growth Characteristics Consistant With MSCs

**Figure 2:**
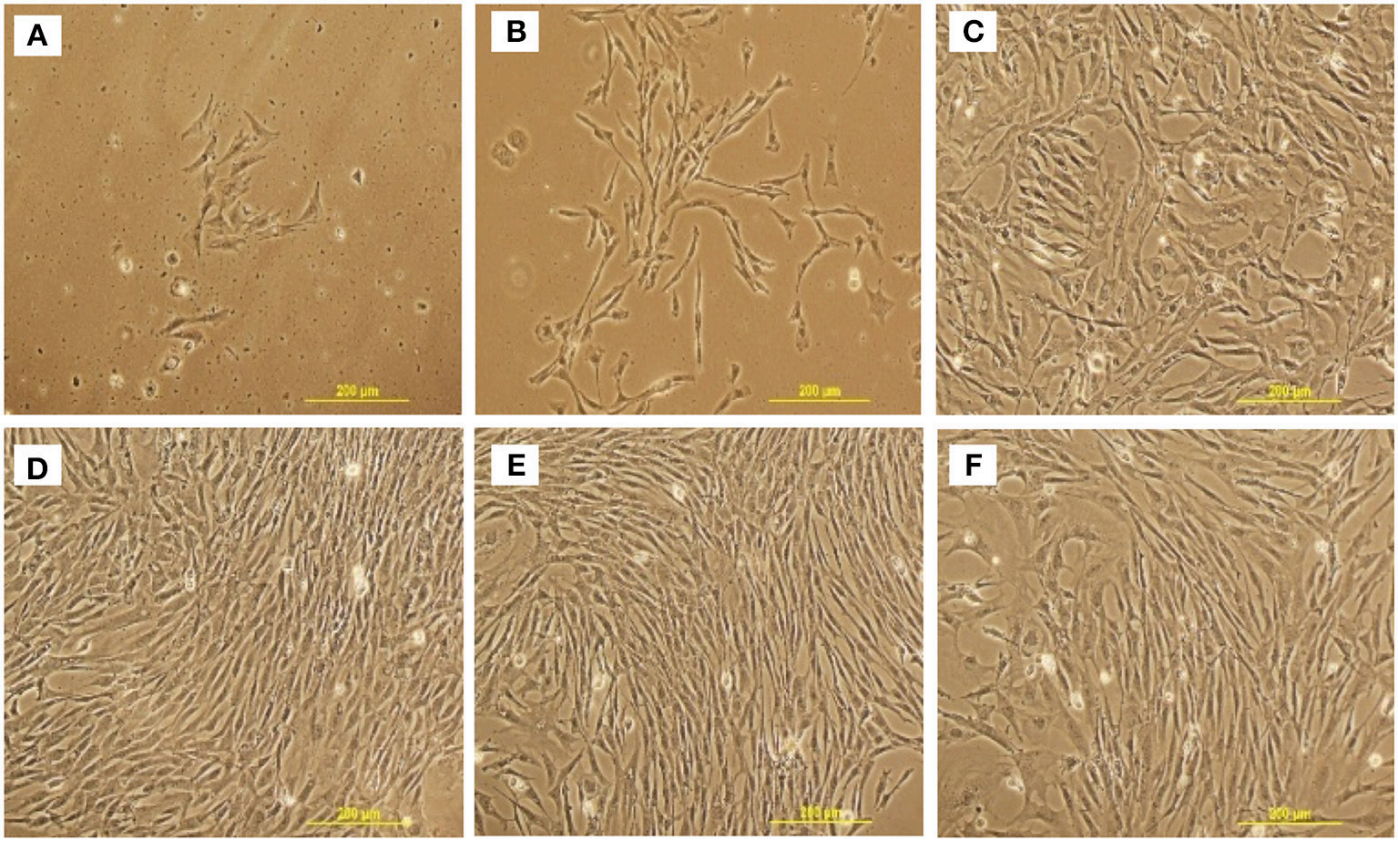
Representative cell morphology at different stages and passage of chicken MSCs isolated from compact bones. **(A)** passage 0; day 4, **(B)** passage 0; day 10, **(C)** passage 2, **(D)** passage 4, **(E)** passage 6, and **(F)** passage 8.

cBMSCs were isolated from 1-day-old broiler compact bones of the tibia and femur (Figure 1). cBMSCs at P0 were round for an initial 2 days and appeared to be spindle-shaped after 3 days of culture (Figure 2A). Cells formed distinct colonies and were passaged 10–12 days after isolation (Figure 2B). Cells were passaged at a ratio of 20,000 cells/cm^2^ for subsequent passage once reaching confluence (Figure 2C). Cells adhered to the plates, were spindle shaped, divided rapidly, and reached confluency within 2–3 days consistent with an MSC phenotype and growth pattern. This rapid proliferation and spindle shaped morphology were consistently observed in subsequent passage 4, 6, and 8 (Figures 2D–F). Cells were passaged up to P12 to observe morphological characteristics. Cells at P4 were used for further analyses of proliferation potential, immunocytochemistry, qRT-PCR, and multilineage differentiation capacity.

### cBMSCs Are Capable of Self-Renewal

**Figure 3:**
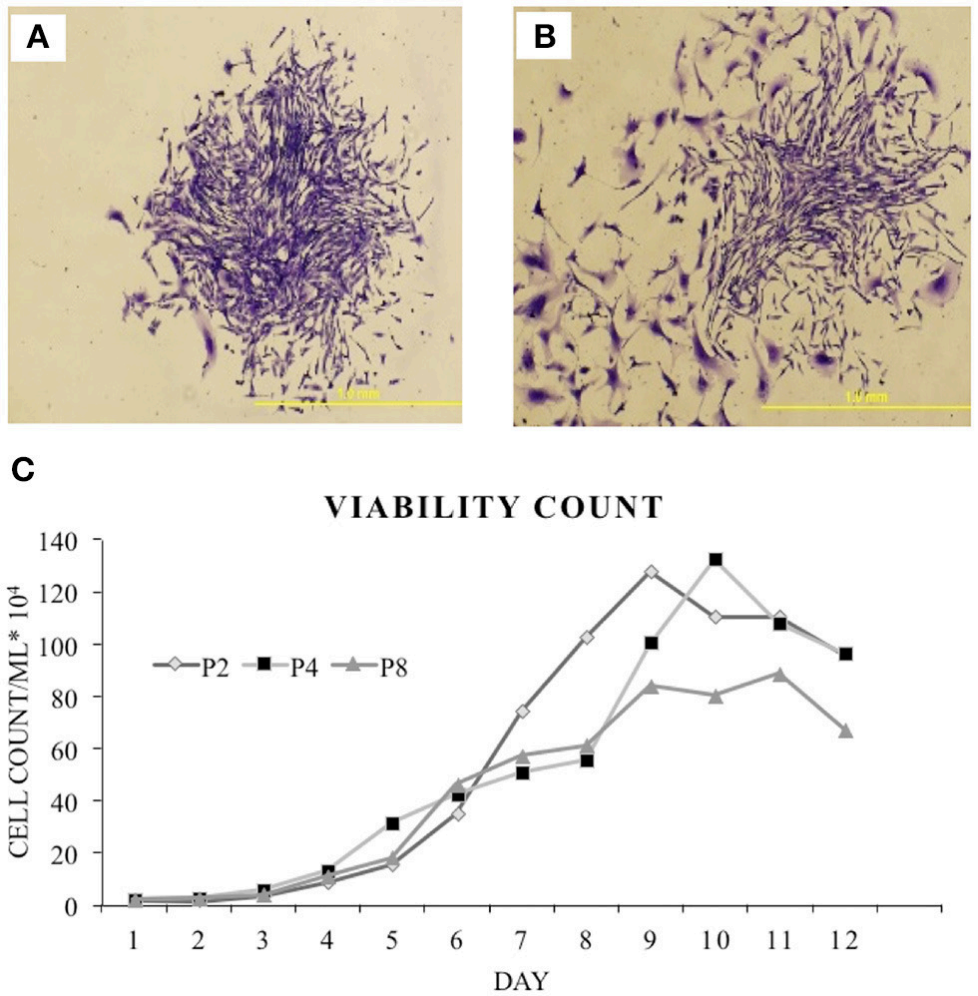
Colony forming assay. Formation of colony from chicken compact bone derived MSCs stained with 1% crystal violet in methanol: **(A)** Passage 4 and **(B)** passage 8. **(C)** Growth curve of the isolated chicken BMSCs. Growth curve of P2, P4, and P8 were all sigmodal in shape that showed latent phase, log phase, and plateau phase.

As cBMSCs have the ability to grow and self-renew, a CFU assay was used to assess the proliferation ability and colony forming potential of isolated cBMSCs. cBMSCs formed distinct colonies of cells when seeded at lower densities, indicating that they have the potential to self-renew (Figure 3). Cells plated at densities of 25 cells/cm^2^, 50 cells/cm^2^, and 75 cells/cm^2^ formed 90, 156, and 209 colonies at P4 (Figure 3A) and 75, 106, and 144 colonies at P8, respectively (Figure 3B).

### Growth Kinetics

A growth kinetics assay with Trypan blue exclusion was performed on cBMSCs by quantifying the number of cells over 12 day period at P2, 4, and 8. Growth and proliferation potential of cBMSCs were similar at P2, 4, and 8. Cells initially had a latent phase of 1–3 days, a logarithmic growth phase for 4–7 days, and reached a plateau phase in about 9–11 days in P2 and 4 and in about 8–9 days in passage 8 (Figure 3C). Thus, this result showed that the cell viability of P8 was overall lower than one of P2 or P4, whereas P2 and P4 showed similar cell viability. PDT of the passage 2, 4, and 8 were 66, 76, and 74 h, respectively. Comparing the growth curve and PDT, cBMSCs showed robust and comparable proliferation rates across passages.

### cBMSC Are Postive for MSC Gene and Protein Expression

**Figure 4:**
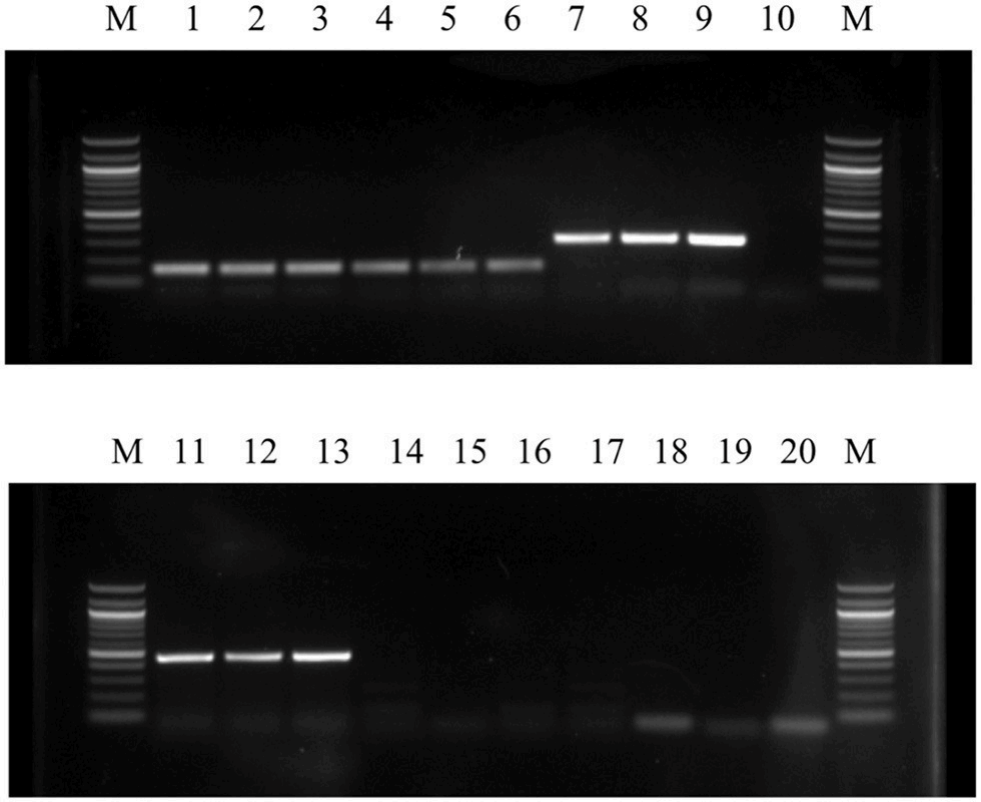
Qualitative expression of cell surface markers of cBMSCs were detected by RT-PCR in all three passages (P2, P4, and P8). High level of expression of cell surface markers CD90, CD105, CD44, CD29, and GAPDH as well as lack of expression of CD 45 and CD34 mRNA in MSCs indicates that the cells were homogenous population or mesenchymal stem cells. M- Marker; Lane 1- P2 GAPDH; Lane 2- P4 GAPDH; Lane 3- P8 GAPDH; Lane 4- P2 CD44; Lane 5- P4 CD44; Lane 6- P8 CD44; Lane 7- P2 CD29; Lane 8- P4 CD29; Lane 9- P8 CD29; Lane 10- Control; Lane 11- P2 CD90; Lane 12- P4 CD90; Lane 13- P8 CD90; Lane 14- P2 CD34; Lane 15- P4 CD34; Lane 16- P8 CD34; Lane 17- P2 CD45; Lane 18- P4 CD45; Lane 19- P8 CD45; and Lane 20- Control.

**Figure 5:**
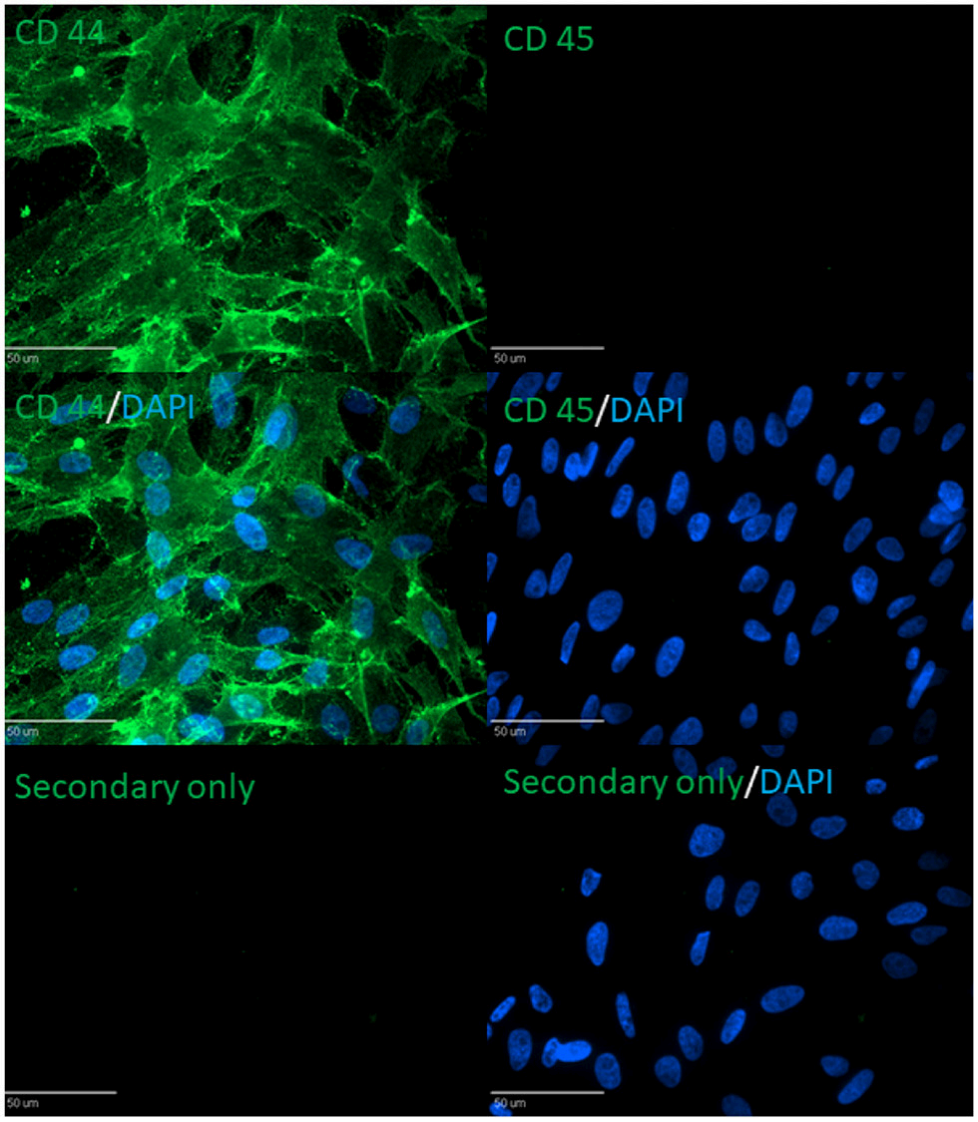
Immunocytochemistry against CD44 (MSC marker) and CD45 (non-MSC marker) in chicken MSC.

cBMSCs were evaluated at P2, P4, and P8 for MSC marker gene expression. cBMSCs at all 3 passages revealed positive results for mRNA transcripts of CD90, CD105, CD73, CD44, and CD29 (Figure 4). CD45 and CD34 mRNA were not expressed in cBMSCs at all 3 passages. In addition, immunofluorescence revealed that chicken cBMSCs expressed CD44 (98.5% ± 1.5) but not CD45 (0%) (Figure 5) further indicating that these cells are cBMSCs (Khatri et al., 2009).

### cBMSCs Demonstrate Osteogenic, Adipogenic and Myogenic Differentiation Potential

**Figure 6:**
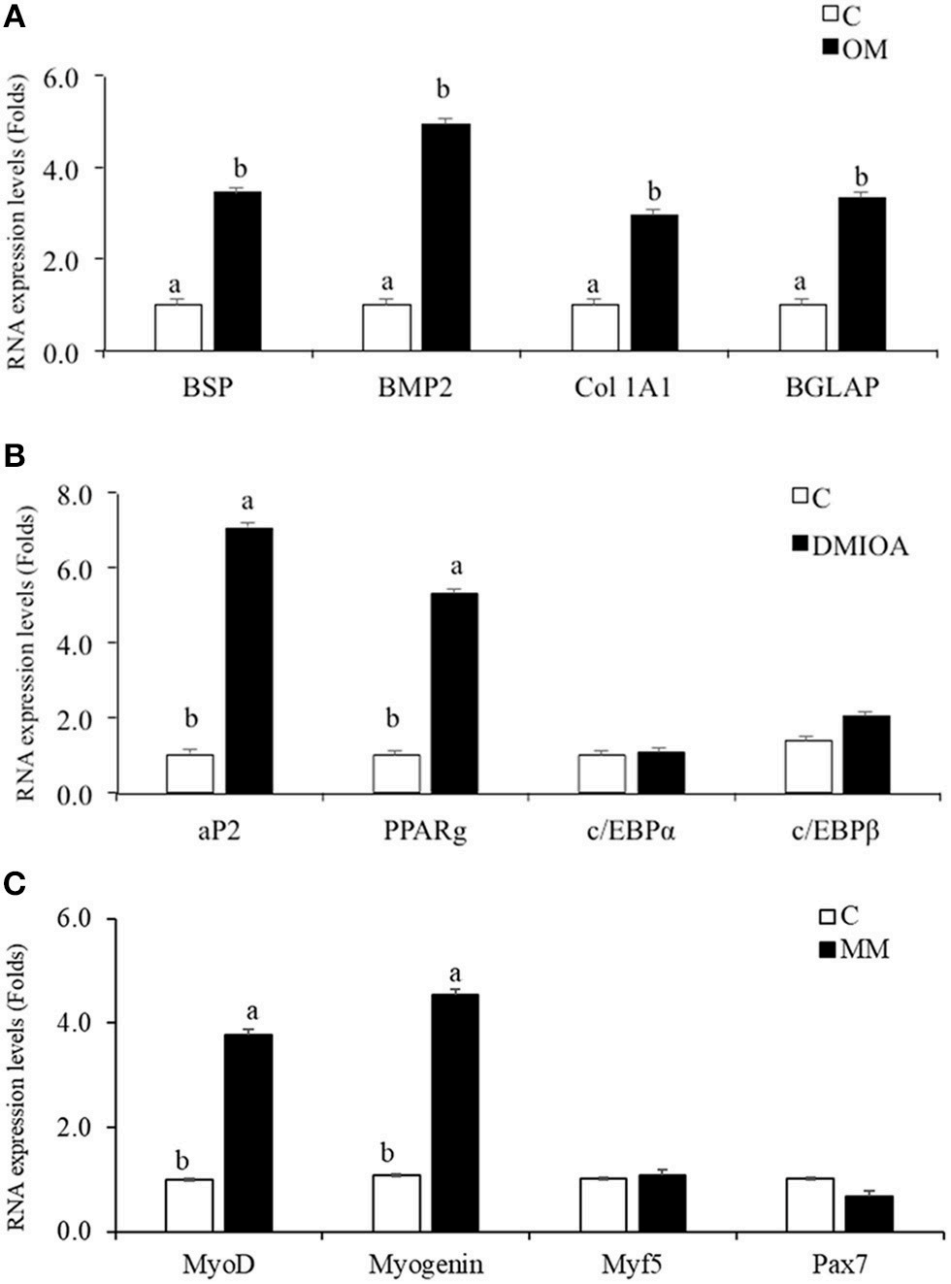
Comparative analysis of lineage differentiation specific gene expression in cBMSc. Relative gene expression of osteogenic, adipogenic, and myogenic markers were analyzed between induced cells and control cells (C) using qRT-PCR. **(A)** BSP, Collagen Type 1 Alpha 2 (Col 1A2), BMP2, and BGLAP mRNA expression were analyzed in cells treated with osteogenic media (OM) (containing DMEM with 10^−7^M dexamethasone (DXA), 10 mM β-glycerophosphate, 50 μg/ml ascorbate, and 5% FBS) and control media (C) for 72 h; **(B)** FABP2, PPARγ, c/EBPα, and c/EBPβ expression were analyzed in cells treated with adipogenic media (AM) (containing 500 nM dexamethasone, 0.5 μM 3-isobutyl-1-methylxanthine, and 20 mg/mL insulin and 300 μM OA) and control media (C) for 48 h **(C)** MyoD, Myogenin, Myf5, and Pax7 mRNA expression were analyzed in cells treated with myogenic media (MM) (containing DMEM, 5% horse serum, 50 μM hydrocortisone, and 0.1 μM dexamethasone) and control media (C) for 72 h. GAPDH was used as a housekeeping gene. Different letters (a,b) indicate significant difference at *P* < 0.05.

cBMSCs treated with specific differentiation media in 6-well plates were harvested for osteogenic, adipogenic, and myogenic gene expression using qRT-PCR. cBMSCs treated with OM expressed higher levels of Runx2, BMP, BSP, and BGLAP mRNA relative to non-treated control cells, indicating osteogenic differentiation of cBMSCs (Figure 6A). cBMSCs treated with adipogenic media expressed a higher level of FABP4 and PPARγ mRNA in comparison to non-treated control cell at 48 h post-treatment. However, c/EBPα, and c/EBPβ were not significantly different between treatments at 48 h post- treatment (Figure 6B). Cells subjected to MM expressed higher levels of Myogenin and MyoD mRNA expression which are early differentiation markers of myogenesis. However, Myf5, and Pax7 were not significantly different between the treated and control cells (Figure 6C). These results demonstrate that cBMSCs are capable of osteogenic, adipogenic, and myogenic differentiation.

### cBMSCs Show Functional Osteogenic and Adipogenic Differentiation Capacity

**Figure 7:**
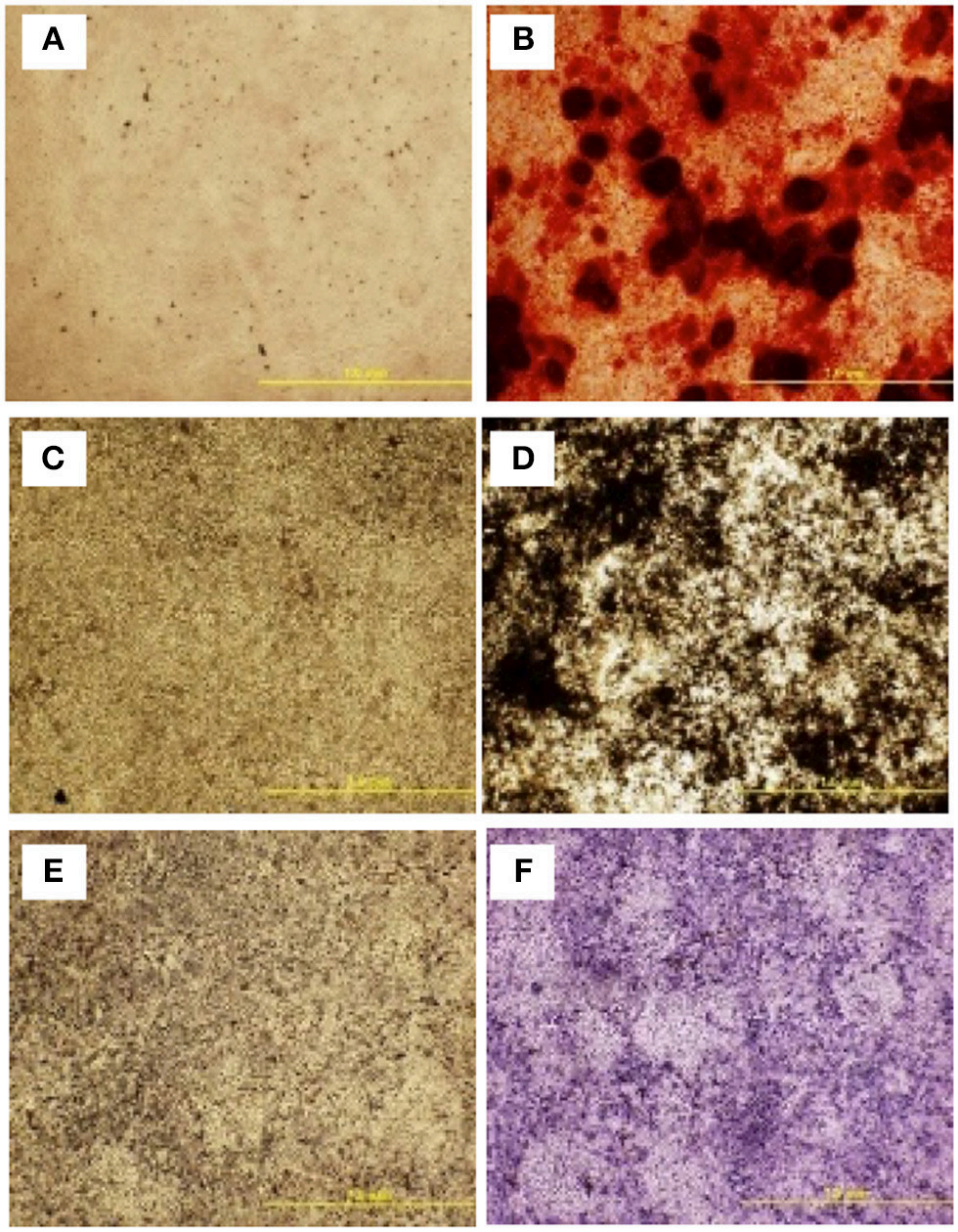
Multilineage differentiation potential of cBMSCs. On confluency, cBMSCs were treated with osteogenic media (OM) containing DMEM with 10^−7^M dexamethasone (DXA) (Sigma Aldrich, MO, USA), 10 mM β-glycerophosphate (Sigma Aldrich, MO, USA), 50 μg/ml ascorbate (Sigma Aldrich, MO, USA), and 5% FBS for osteogenic induction. Cells cultured in DMEM basal media with 10% FBS were used as negative control. Both cells were stained with Alizarin red stain and Von Kossa stain at day 14. Alizarin Red stain in **(A)** control cells **(B)** OM treated cells. Von Kossa stain in **(C)** control cells and **(D)** OM treated cells. Alkaline Phosphate assay in **(E)** control cells and **(F)** OM treated cells.

**Figure 8:**
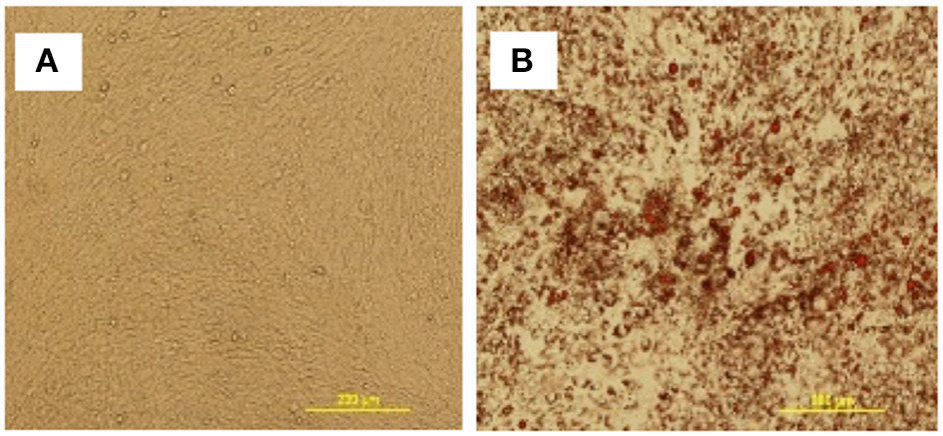
Adipogenic differentiation of cBMSCs. cBMSCs were treated with adipogenic cocktail (DMI containing 500 nM dexamethasone, 0.5 mM 3-isobutyl-1-methylxanthine, and 20 mg/mL insulin) and 300 μM of OA (DMIOA) when cells were confluent. Cells cultured in 10% DMEM were used as negative control. Differentiation of adipocyte was detected by Oil Red O stain in **(A)** control cells vs. **(B)** induced cells at 96 h.

To further assess the multipotent differentiation capacity of cBMSCs, cells were differentiated under osteogenic, and adipogenic conditions. To first assess osteogenic differentiation potential, MSCs were treated with OM for 2 wk. At the end of wk1 and wk2, calcification deposits of cells treated with OM were detected with cells staining positive for Alizarin Red and Von Kossa, both of which were negative in control cells not treated with OM differentiation media. Differentiated cells stained with Alizarin red were bright orange-red in color (Figure 7B), whereas undifferentiated cells were not (Figure 7A). Differentiated cells stained with Von Kossa stain showed brown to black mineralized deposits, indicating increased mineralization of differentiated cells (Figures 7C,D). Cells treated with OM showed positive alkaline phosphatase activity compared to untreated cBMSCs. Undifferentiated cBMSCs were colorless or faint blue (Figure 7E), whereas differentiated cBMSCs stained dark blue-violet (Figure 7F). Cells treated with adipogenic media displayed an adipocyte phenotype with the appearance of cytoplasmic lipid vacuoles detected by oil red O staining (Figure 8B). The control cells did not show any adipocyte formation (Figure 8A).

## Discussion

In this study, we have for the first time characterized MSCs from compact bones of broiler chickens. MSCs isolated from compact bones displayed spindle-shaped cells that proliferate rapidly and arrange in a whirlpool pattern similar to MSCs isolated from murine compact bones (Zhu et al., 2010; Short and Wagey, 2013). Cells were able to self-renewal forming colonies from single cells and display osteogenic, adipogenic, and myogenic differentiation (Dominici et al., 2006). This study also demonstrated that cBMSCs possessed the critical 3 MSC defining characteristics of rapid proliferation, adherence to plastic, and multilineage differentiation as described by ICST (Dominici et al., 2006).

The growth curve of the cBMSCs isolated from compact bones in this study is similar to the growth curve of the MSCs isolated from non-compact bone marrow of chicken (Khatri et al., 2009; Bai et al., 2013). In the present study, cells had a latency phase of 1–3 days with a logarithmic phase and a plateau phase in about 7–8 days. PDT of the cBMSCs were 66, 76, and 74 h for P2, P4, and P6, respectively, which was different from the PDT of MSCs isolated from mouse which was 20 h for primary culture and 80 h at passage 3 (Nadri and Soleimani, 2007). Also, in guinea pig, PDT of cells isolated from bone marrow were 62.9, 65.6, and 91.4 h at P 2, 5, and 8, respectively (Aliborzi et al., 2016). These reports indicate that the growth potential of the MSCs could vary depending on the passage rate as well as tissue source of MSCs isolation and species of origin.

cBMSC demonstrated multilineage differentiation potential when cultured in osteogenic, adipogenic, and myogenic differentiation conditions. cBMSCs treated with OM promoted osteogenic differentiation of MSCs by increasing osteogenic gene transcripts. Similarly, in previous reports, treatment of MSCs with OM resulted in osteogenic differentiation of MSCs isolated from bone marrow in poultry (Bhuvanalakshmi et al., 2014) and in humans (Noth et al., 2002; Klepsch et al., 2013). Osteogenic differentiation of cells was characterized by staining the treated cells with Alizarin Red, Von Kossa, and ALP. These cytochemistry tests are routinely used to characterize the osteogenic differentiation of MSCs (Donato et al., 2016; Shi et al., 2016). ALP, Alizarin Red and Von Kossa stain both indicate mineralization of MSCs through osteogenic differentiation (Parhami et al., 1997; Gregory et al., 2004). Differentiated osteoblasts expressed a high ALP activity compared to undifferentiated MSCs which expressed very weak activity. Undifferentiated MSCs do not have extracellular calcium deposits, whereas differentiated osteoblasts do. Calcium deposit is an excellent way to detect if there is osteogenic differentiation of MSCs when treated with OM (Bellows et al., 1986; Wang et al., 2006). Silver ions in Von Kossa stain reacts with anions (phosphates, sulfates, or carbonates) of calcium salts in the cells and the reduction of silver salts forms a brown to black stain. However, Von Kossa stain alone cannot unequivocally confirm osteogenic differentiation of cells and calcium deposits as AgNo_3_ could be displaced by SO4 ions in any metal (Bonewald et al., 2003). Another test done to confirm calcium deposits along with Von Kossa stain is the Alizarin Red test. Alizarin Red reacts with calcium cations to form an orange-red chelate thus confirming the deposits of Ca in cells (Wang et al., 2006). Further osteogenic differentiation was confirmed in cBMSCs by analyzing key osteogenic genes using qRT-PCR. BGLAP, BSP, BMP2, and ColA1 mRNA was highly expressed in cells treated with OM which indicates osteogenic differentiation capacity of MSCs isolated in this study. Treatment with OM induced similar osteogenic gene expression and expressed positive ALP, Alizarin Red, and Von Kossa stain in MSCs derived from bone marrow of chicken (Bai et al., 2013), mouse (Nadri and Soleimani, 2007), bovine (de Moraes et al., 2016), and human (Kulterer et al., 2007; Honda et al., 2013). In this study, differentiated cells transformed from elongated to shorter cuboidal cells and formed mineralized nodules, which was also reported in MSCs derived from bone marrow of chickens (Khatri et al., 2009).

Adipogenic differentiation of cBMSCs was induced by treating cells with adipogenic cocktail DMI and OA in this study. The characteristics and molecular mechanism of adipocyte differentiation in murine preadipocyte cell line 3T3-L1 has been extensively studied (Ntambi and Young-Cheul, 2000). It has been reported that preadipocytes after differentiation from MSCs stay in growth arrest stage, which reenters into the cell cycle and undergoes mitotic division in response to an adipogenic cocktail, which then terminally differentiates into adipocytes (Tang et al., 2003). However, in human adipose precursor cells derived from adipose tissue and bone marrow, MSCs do not undergo cell division during differentiation (Entenmann and Hauner, 1996; Lehmann et al., 1997). The exact mechanism of adipogenic differentiation by addition of DMI and AO in chicken MSCs is not known. In our study, addition of DMI and OA induced adipogenic differentiation of cBMSCs which was detected by accumulation of lipid vacuole within the cells and an increase in the master regulator of adipogenesis, PPARγ. This is in agreement with human (Tontonoz et al., 1994; Neubauer et al., 2004; Scott et al., 2011) and mouse (Scott et al., 2011) adipogenic differentiation studies. Similarly, DMI and OA increased PPARγ, and FABP4 mRNA expression during an early stage of adipogenesis in pre-adipocyte cells isolated from abdominal adipose tissue of 10-day old broiler chick (Matsubara et al., 2005). However, no increase in c/EBPα and c/EBPβ mRNA expression at 48 h in cMSCs has been observed in the current study. This result is not in agreement with studies with mouse 3T3-L1 cells and hen preadipocytes (Ntambi and Young-Cheul, 2000; Tang et al., 2003; Regassa and Kim, 2013). Since c/EBPβ is an early adipogenic transcription factor, this gene would have been expressed before 48 h. Typically, c/EBPα and PPARγ expression increases during adipogenesis because of the positive feedback loop between c/EBPα and PPARγ (Wu et al., 1999). There are some differences in adipogenic mechanisms among species and cell sources. Hen preadipocytes showed significant c/EBPα and c/EBPβ mRNA expression at 48 h during adipogenic differentiation (Regassa and Kim, 2013). Further studies are necessary to understand uniqueness of cMSCs for adipogenic mechanisms. Myogenic regulatory factors orchestrate the differentiation of MSCs into muscles cells. MyoD and Myogenin are believed to be early differentiation markers of myogenic differentiation, and Myf5 and MRF4 are believed to regulate terminal differentiation and cell fusion (Perez-Serrano et al., 2017). Similar to muscle development, MyoD and Myogenin are main muscle-specific transcription factors that are expressed during myogenic differentiation of MSCs (Gang et al., 2004, 2008). In this study, cBMSCs expressed higher MyoD and Myogenin mRNA levels when subjected to myogenic media. This indicates that cBMSCs are capable of differentiating into osteogenic, myogenic, and adipogenic lineages when subjected to appropriate differentiation conditions.

Because availability of stem cell specific markers is limited in poultry, researchers have to rely on reports of cell surface markers in mammalian species. Use of markers to verify MSC identity is an important quality control step to reduce experimental variability and obtain a homogenous population of MSCs. Our study detected the presence of mesenchymal cell surface markers CD90, CD105, CD73, CD44, and CD29 and lack of hematopoietic cell surface markers CD45 and CD34 mRNA expression at all passage studies. Furthermore, immunocytochemistry confirmed that chicken MSCs were positive for the MSC marker CD44 and negative for the hematopoietic stem cell marker CD45. This demonstrates that the characteristic immunophenotype of cBMSCs is consistent with previously reported chicken MSCs (Khatri et al., 2009; Bai et al., 2013; Intarapat and Stern, 2013).

Despite significant progress in our understanding of MSC biology based on human and murine cells, much of the information pertaining to avian MSCs remains poorly defined including identity and functionality. In the present study, we showed that MSCs can be isolated from the compact bone of day-old broilers, cultured, and characterized. The adherence, morphology, differentiation potential, and specific markers are comparable with the MSCs derived from other source and animals, making them a suitable model for various research applications. Establishment of primary MSC culture can have a huge potential impact on our understanding of chicken development and nutrition with increased ease of availability and a better defined and characterized isolation and culture conditions. Chicken compact bone derived MSCs could be used as an ideal stem cell source for various biological research because of its easy purification, amplification, multipotency, and maintenance. cBMSCs can be a cell culture model in avian species to understand osteogenic, adipogenic, and myogenic differentiation mechanism exerted by different bioactive/nutrient compounds, which can promote skeletal health, muscular growth, fat development, and efficient feed utilization in poultry.

## Author Contributions

All authors listed have made a substantial, direct and intellectual contribution to the work, and approved it for publication. WK and FW conceived and designed this study. RA contributed to isolation of cBMSCs, cell differentiation, qRT-PCR, PCR for cell surface markers, and data analyses. CC and EW contribute to cell isolation, immunocytochemistry, and data analyses. The paper was written through contribution and critical review of the manuscript by all authors (RA, CC, EW, FW and WK).

### Conflict of Interest Statement

The authors declare that the research was conducted in the absence of any commercial or financial relationships that could be construed as a potential conflict of interest.

## Abbreviations

ALP: Alkaline Phosphatase Assay
BMP: Bone Morphogenic Protein
BSP: Bone Sialoprotein
BGLAP: Bone Carboxyglutamate Protein
MSCs: Mesenchymal Stem Cells
cBMSCs: Chicken Compact Bone Derived Mesenchymal Stem Cells
OA: Oleic Acid
DMI-Dexamethasone, 3, isobutyl-1-methylxanthine: and Insulin
FBS: Fetal Bovine Serum
DMIOA, Dexamethasone, 3-isobutyl-1-methylxanthine, Insulin: and Oleic Acid
qRT-PCR: Quantitative Real-Time Reverse Transcriptase Polymerase Chain Reaction
FABP4: Fatty Acid Binding Protein 4
PPARγ2: Peroxisome Proliferator-Activated Receptor gamma
DMEM: Dulbecco's Modified Eagle's Medium
SAS: Statistical Analysis System.
